# An Overview on the Therapeutics of Neglected Infectious Diseases—Leishmaniasis and Chagas Diseases

**DOI:** 10.3389/fchem.2021.622286

**Published:** 2021-03-12

**Authors:** Brindha J, Balamurali M. M, Kaushik Chanda

**Affiliations:** ^1^ Division of Chemistry, School of Advanced Sciences, Vellore Institute of Technology, Chennai, India; ^2^ Department of Chemistry, School of Advanced Sciences, Vellore Institute of Technology, Vellore, India

**Keywords:** chagas, leishmaniasis, infectious disease, therapeutics, drugs

## Abstract

Neglected tropical diseases (NTDs) as termed by WHO include twenty different infectious diseases that are caused by bacteria, viruses, and parasites. Among these NTDs, Chagas disease and leishmaniasis are reported to cause high mortality in humans and are further associated with the limitations of existing drugs like severe toxicity and drug resistance. The above hitches have rendered researchers to focus on developing alternatives and novel therapeutics for the treatment of these diseases. In the past decade, several target-based drugs have emerged, which focus on specific biochemical pathways of the causative parasites. For leishmaniasis, the targets such as nucleoside analogs, inhibitors targeting nucleoside phosphate kinases of the parasite’s purine salvage pathway, 20S proteasome of *Leishmania*, mitochondria, and the associated proteins are reviewed along with the chemical structures of potential drug candidates. Similarly, in case of therapeutics for Chagas disease, several target-based drug candidates targeting sterol biosynthetic pathway (C14-ademethylase), L-cysteine protease, heme peroxidation, mitochondria, farnesyl pyrophosphate, etc., which are vital and unique to the causative parasite are discussed. Moreover, the use of nano-based formulations towards the therapeutics of the above diseases is also discussed.

**GRAPHICAL ABSTRACT:**
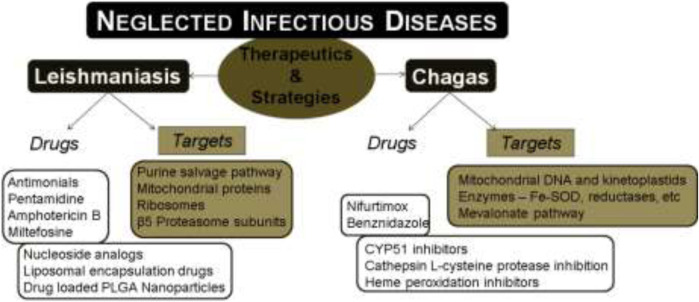


## Introduction

Neglected tropical diseases (NTDs) include a group of diverse infectious diseases that are more prevalent in the tropical and subtropical regions of the world. These diseases are more associated with the poverty zones that have limited health facilities. Moreover, these infections are biologically diverse with 20 different diseases under the list of NTDs including Buruli ulcer, Chagas disease, dengue, chikungunya, dracunculiasis (guinea-worm disease), echinococcosis, foodborne trematodiases, human African trypanosomiasis (sleeping sickness), leishmaniasis, leprosy (Hansen’s disease), lymphatic filariasis, mycetoma, chromoblastomycosis and other deep mycoses, onchocerciasis (river blindness), rabies, scabies and other ectoparasites, schistosomiasis, soil-transmitted helminthiases, snakebite envenoming, taeniasis/cysticercosis, trachoma, and yaws (endemic treponematoses). For the past few decades, many pharmaceutical companies have been supportive towards combating these diseases and thereby a significant portion of the economic burden on developing countries was reduced. There are five different strategies adopted by WHO to manage these NTDs: i) preventive chemotherapy, ii) intensified disease management, iii) vector control, iv) neglected zoonotic diseases, and v) improved water and sanitation. In the past, several programs to alleviate NTDs were implemented to assist in controlling and eliminating these diseases completely. Many targets proposed in the roadmap of WHO in 2012 have not been achieved to date, though progress is being made to overcome the global impact of NTDs. Recently, the new NTD roadmap for the years 2021–2030 was proposed by WHO (WHO, 2020). WHO has revealed the challenges ahead due to the prevailing COVID-19 pandemic that can hinder the delivery of essential health products for NTDs.

The research on new therapeutic strategies for NTDs focuses on a combination of existing or repurposed drugs, to enhance the efficacy/bioavailability of the available therapeutics; however, the discovery of new drugs also plays a major role in drug discovery for NTDs. Presently available therapies were proved to be toxic and require a longer duration of treatment as compared to the rate of disease progression, along with the development of drug resistance particularly in people infected with NTDs like Chagas disease and leishmaniasis. The above two NTDs are known for the highest mortality rate among others (Hotez et al., 2007).

There are two major ways to identify compounds for these diseases: phenotypic and target-based approaches. The phenotypic approach involves the development of a drug without any knowledge about its target or specific function against the disease (Moffat et al., 2017). In this case, the entire organism, including the pathways/targets, needs to be screened against the drug. The phenotypic approach has been successful in drug discovery and it involves the evaluation of various chemicals against the phenotypes or characteristics observed in an organism (Moffat et al., 2017). But the former lacks understanding of the molecular mechanisms that lead to empirical analysis and delays the progress in obtaining the best drug candidate (Swinney, 2013). The phenotypic approach has the advantage of addressing the problems like cell uptake/efflux and membrane permeability and counterscreening mammalian cells while identifying drug candidates that are active against the whole cell (Borsari et al., 2019). The high-throughput screening mode has enabled the screening of large libraries of drug candidates against whole-cell parasites. In the target-based approach, the first step involves the identification of possible molecular targets that are significantly involved in the disease (Swinney, 2013). This is followed by designing small molecules that interfere with the potential targets in the causative agent (parasite/bacteria/virus) which are absent/different from those present in the infected host system. This approach is rational and exploits information on the genetics, chemistry, and computational sources, for drug discovery. It also quantitatively measures the drug toxicity/dosages (Capdeville et al., 2002; Durieu et al., 2016). The major drawback in the target-based approach (Lai et al., 2002) is the low productivity caused by poor disease linkages and the molecular complexities that are involved in drug’s mode of action (Brown, 2007; Swinney and Anthony, 2011), although in ideal cases, drug discovery can progress through knowledge gained by empirical methods that relate the drug to the molecular mechanisms of action and the involved phenotype to obtain more reliable targets/hypotheses (Garcia-Calvo et al., 2005; Swinney, 2013). There are only a few validated drug targets known against the parasitic diseases and in case of some registered drugs, either the mode of action is poorly understood or it involves various targets (Gilbert, 2013). The correct balance between phenotypic and target-based approaches could possibly lead to successful drug discovery.

Both leishmaniasis and Chagas disease are caused by infectious parasites which are fatal if ignored and untreated. In both cases, the causative parasite shows many unique potential targets in the biochemical machinery. This includes some pathways or targets like the purine/pyrimidine salvage pathways (Bora and Jha, 2020), nucleoside analogs, kinetoplastid proteasomes, mitochondria, etc. There are various potential drug targets available for the treatment of NTDs and intense research is still progressing in this area. The limitations with current therapeutics like toxicity and resistance have made the discovery of novel drugs against leishmaniasis and Chagas disease more essential.

In this review, we discuss various available drugs, emerging drug targets, and drug candidates identified by target-based approach against the two NTDs, leishmaniasis and Chagas diseases. We have also summarized the various therapeutic strategies that have been adopted in the past and present for the treatment of these two infectious neglected diseases.

## Leishmaniasis

Leishmaniasis is one of the infectious NTDs caused by a protozoan parasite vector of genus *Leishmania* that is transmitted to humans through an infected blood-sucking sandfly. There are about 21 reported species of *Leishmania* that causes infections in humans and are endemic in the regions of Asia, Africa, America, and also southern parts of Europe. In leishmaniasis, the causative parasite Leishmania species displays a digenetic life cycle, which includes the extracellular motile promastigote, present in the female sandfly vector of the genus Phlebotomus (throughout Africa/Asia) or Lutzomyia (in America) (Matlashewski, 2001), and intracellular nonmotile amastigote that replicates within the phagolysosomes of host human macrophages. For instance, *P. argentipes* and *P. Salehi* were proven to be the sandfly vectors that causes leishmaniasis in India; *P. celiae, P. martini, P. saevus, P. sergenti, P. duboscqi, P. longipes, P. pedifer,* and *P. sergenti* are some of the sandfly vectors of *Phlebotomus species* that cause leishmaniasis in Ethiopia (Maroli et al., 2013); *Lutzomyia longipalpis* was the major vector in the north and south Americas to cause leishmaniasis infections (Lainson and Rangel, 2005). Leishmaniasis infections lead to different kinds of diseases based on the species causing the infection (Pearson and Sousa, 1996; Ghedin et al., 1997). In humans, leishmaniasis is reported in three different forms: visceral, mucocutaneous, and cutaneous (Modabber et al., 2007). Visceral leishmaniasis, also called Kala-Azar caused by *Leishmania donovani* or *L. chagasi*, is a serious form of infection, manifested with skin darkening, weight loss, fever, liver/spleen enlargement, and emaciation (Matlashewski, 2001). Visceral leishmaniasis is a chronic infection, associated with high mortality and morbidity. Around 10% of the cured Kala-Azar patients develop post-Kala-Azar dermal leishmaniasis, which is chronic with disfigurement of cutaneous nodules (Adams et al., 2013). Dermal leishmaniasis predominantly occurs as localized cutaneous leishmaniasis along with other aggressive forms like diffused cutaneous leishmaniasis, mucosal leishmaniasis, and cutaneous leishmaniasis. They cause several lesions/disfigurement affecting the psychological well-being of the patients (Alvar et al., 2012). Cutaneous leishmaniasis involves the development of large open sores/lesions from several small lumps at the site of insect bite that eventually heals on its own over an extended period of several months (Matlashewski, 2001; Modabber et al., 2007). Diffused cutaneous leishmaniasis is another form of the disease where lesions are developed over a larger part of the body that resolves only with treatment (Matlashewski, 2001). Mucocutaneous leishmaniasis starts initially as cutaneous leishmaniasis and later spreads to mucous membranes of pharynx, mouth, and nose, depletes the tissues, and causes excessive damage to the face, along with leprosy kind of stigma and impairment of breathing in critical cases (Toledo et al., 2013; Pal et al., 2017; Cruz et al., 2019). Psychiatric disorder is associated with patients with extensive lesions that are formed due to cutaneous leishmaniasis (Yanik et al., 2004). These patients were found to have a low quality of life with symptoms of depression, high anxiety, and low body image satisfaction (Chahed et al., 2016). The currently available antileishmanial drugs to treat leishmaniasis are associated with several side effects, toxicity, and drug resistance.

### Standard Anti-Leishmanial Drugs

**FIGURE 1:**
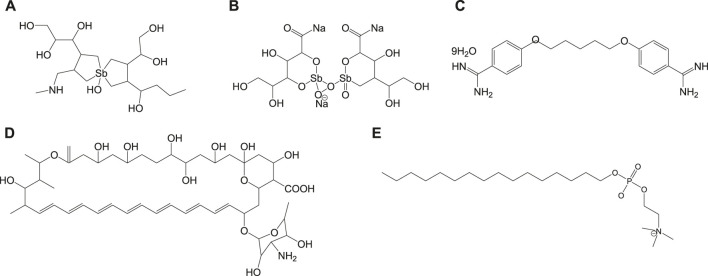
Chemical structure of standard antileishmanial drugs.

Currently used antileishmanial drugs include pentavalent antimonials, pentamidine, amphotericin B, and miltefosine and are known to target different metabolic pathways as discussed in Table 1. Figure 1 depicts the chemical structures of standard drugs reported in the literature to treat leishmaniasis namely: **1a** (meglumine antimoniate or Glucantime®) and **1b** (sodium stibogluconate or Pentostam®), **1c** (pentamidine), **1d** (amphotericin B), and **1e** (miltefosine). To date, the *pentavalent antimonials* are used for the treatment of all the three clinical forms of leishmaniasis. Some examples of pentavalent antimonials include **1a** and **1b** (Goodwin, 1995) (Figure 1). During the last century, antimony (III) potassium tartrate (tartar emetic) was used in the treatment of mucocutaneous leishmaniasis, which was then proved to be effective for visceral leishmaniasis in Africa (Cole, 1944), followed by its interruption in clinical usage due to severe side effects. In the 1940s, less toxic pentavalent antimony [Sb(V)] complexes (Berman, 1997) that includes **1a** and **1b** were reported to be more effective (20 mg of Sb/kg/day for 20–30 days). There are two main models that describe the mechanism of action of these pentavalent antimonials in the treatment of leishmaniasis. The first model involves a prodrug concept, in which Sb(V) gets reduced to Sb(III), as observed in several *in vivo* studies (Burguera et al., 1993; Shaked-Mishan et al., 2001), to exhibit antileishmanial activity. Studies have suggested the involvement of thiols like trypanothione, cysteine, and cysteinylglycine or glutathione preferably in the amastigotes (dos Santos Ferreira et al., 2003), along with the involvement of enzymes that are specific to parasites like thiol-dependent reductase/antimoniate reductase (Zhou et al., 2004). In the second model, Sb(V) itself was shown to possess anti-leishmanial activity, by forming complexes with ribose containing molecules and by inhibiting the type I DNA topoisomerases of *L. donovani* (Chakraborty and Majumder, 1988; Lucumi et al., 1998; Demicheli et al., 2002; Walker and Saravia, 2004)*.* Due to affordability and high cure rate, these pentavalent antimonials have found their use as the first-line therapeutics for leishmaniasis.

**TABLE 1:** Standard drugs against leishmaniasis disease.

Standard drug	Mechanism of action	Advantages	Disadvantages	References
Pentavalent antimonials, meglumine antimoniate, and sodium stibogluconate (**1a**)	Not well known, but two model systems are: Prodrug model- conversion of Sb(V) to toxic Sb(III) and intrinsic Sb(v) activity: By complex formation with ribose/inhibition of type I DNA topoisomerase	Considered as first-line drugs	•Daily parenteral administration	(Berman (1997); dos Santos Ferreira et al. (2003); Walker and Saravia (2004); Zhou et al. (2004)
			•Drug resistance	
			•Side effects include nausea, vomiting, weakness and myalgia, abdominal colic, diarrhea, skin rashes, hepatotoxicity, and cardiotoxicity	
Pentamidine (**1c**), pentamidine mesylate, and pentamidine isethionate	Not known; drug entry through polyamine/arginine transporters	Second line of defense	Drug resistance which may involve mitochondria/ABC protein PRP1	Basselin et al. (1996); Kandpal and Tekwani (1997); Mukherjee et al. (2006); Coelho et al. (2007)
Amphotericin B (**1d**)	Channel/pore formation on interaction with membrane sterol	•First line of treatment replacing the antimonials against visceral leishmaniasis	Toxic with serious side effects: renal impairment, anemia, fever, malaise, and hypokalaemia	Hartsel and Bolard (1996); Neumann et al. (2009); Cohen (2010); Hamill (2013)
		•Nephrotoxicity overcome by amphotericin B formulations: liposomes, nanoparticles, and emulsions		
Miltefosine (**1e**)	Apoptosis like cell death, targeting lipid metabolism, mitochondrial, and immunomodulatory effects	As monotherapy to treat both cutaneous and visceral leishmaniasis	•Side effects, mild-to-moderate gastrointestinal problems and mild nephrotoxicity	Jha et al. (1999); Sundar and Olliaro (2007); Dorlo et al. (2012a); Dorlo et al. (2012b)
			•Long half-life and teratogenicity limit the administration of miltefosine in pregnant women	

*Pentamidine* (**1c**) was previously available in two forms: pentamidine methanesulfonate and pentamidine isethionate salt (Ghorbani and Farhoudi, 2018). Currently, pentamidine methane sulfonate formulations are not available but pentamidine isethionate is still in use. Treatment of cutaneous leishmaniasis using pentamidine mesylate and pentamidine isethionate is reported with nearly 90% cure rate without relapse of infection (Lai et al., 2002). No serious side effects except for respiratory tract problems were observed in patients treated with pentamidine isethionate. **1c** of dosage, 2 mg/kg every other day for a total of seven injections, was proved to be effective against 80% of patients (in Colombia) for the treatment of cutaneous leishmaniasis (Soto-Mancipe et al., 1993). On comparison of **1c** with **1a** against the treatment of leishmaniasis caused by *L. braziliensis*, **1c** was less effective than **1a** (in Peru) (Andersen et al., 2005). Although its mechanism of action is not well known, the **1c** compounds are found to enter *L. donovani via* the transporters of arginine and polyamine (Kandpal and Tekwani, 1997). This drug was reported to accumulate in the mitochondria (Mukherjee et al., 2006), by decreasing mitochondrial membrane potential and inhibit mitochondrial topoisomerase II (Basselin et al., 1996). The drug resistance in case of **1c** was due to more drug efflux and reduced uptake of the drugs through the involvement of intracellular ABC protein PRP1 (pentamidine resistance protein 1 (Coelho et al., 2007). *Amphotericin B* (**1d**) is another choice of treatment for leishmaniasis in cases where resistance to pentavalent antimonials was observed. **1d** is a polyene antifungal compound (fungizone, C_47_H_73_NO_17_) that targets sterol pathway, mainly ergosterols present in the cell membrane of the parasite (Purkait et al., 2012). The mechanism of action of **1d** involves the formation of pores or channels on the lipid bilayer membranes of the targeted host cells (Baginski et al., 1997; Neumann et al., 2009). The channels or pores thus created by the membrane active drug **1d** lead to influx of ions/solutes, thereby causing cell death. Membrane sterols are necessary for **1d** to interact and form these channels/pores on the membrane (Hartsel and Bolard, 1996; Cohen, 2010). There are different formulations of **1d**, for instance, amphotericin B lipid complex or ABLC (Abelcet), liposomal amphotericin B or L-AmB (AmBisome), amphotericin B colloidal dispersion, or ABCD (amphocil and amphotec) (Hamill, 2013). ABLC is composed of amphotericin B bound to two lipids, namely, L-α-dimyristoyl phosphatidylcholine (DMPC) and L-α-dimyristoyl phosphatidylglycerol (DMPG), and has a therapeutic dosage of 5 mg/kg/day. ABLC is the largest among these three lipid molecules and was shown to be less effective than the amphotericin B deoxycholate in the stimulation of proinflammatory signaling molecules (Arning et al., 1995; Simitsopoulou et al., 2005). L-AmB was proven to be successful against visceral leishmaniasis, with a dosage level of 3–6 mg/kg/day (Davidson et al., 1991; Boswell et al., 1998). With a single dose of L-AmB, the concentration of the drug in plasma exceeds the concentration achieved by the conventional amphotericin B deoxycholate (Meyerhoff, 1999; Walsh et al., 2001), whereas in case of ABCD (with amphotericin B and cholesteryl sulfate, 3-4 mg/kg/day dosage), the concentration in plasma was less than that maintained by the conventional drug amphotericin B deoxycholate (Herbrecht et al., 1999; Hamill, 2013). Liposomal amphotericin B is an FDA-approved drug for the treatment of visceral leishmaniasis, which is usually administered through intravenous infusion.

*Miltefosine* (**1e**) or hexadecylphosphocholine (Impavido^®^) is an alkyl phospholipid and an oral drug that is used for the treatment of all three forms of leishmaniasis. These compounds are phosphocholine analogs used for oral treatment against visceral leishmaniasis disease (Sundar and Olliaro, 2007; Dorlo et al., 2012b). **1e** which works by targeting the cell membrane proteins/signaling pathways of the parasites induces cytotoxicity, ultimately leading to apoptosis. With the intake of 100 mg of miltefosine/day (around 2.5 mg/kg of body weight/day) for a month, high efficacy with 97% cure rate was reported (Jha et al., 1999). However, in the same study, side effects like mild-to-moderate gastrointestinal problems were observed. The mechanism of action of **1e** is not very clear, although different modes of actions have been proposed by several studies. Induced apoptosis-mediated cell death of the parasites (*L. donovani*), based on nuclear DNA condensation and fragmentation, was proposed (Verma and Dey, 2004). **1e** was given as a monotherapy to treat cutaneous or visceral leishmaniasis with a dosage of 2.5 mg/kg/day for a total of 28 days (Dorlo et al., 2012b). **1e**, initially an anticancer drug, was reported to have similar molecular modes of action against both the *Leishmania* spp. and the cancer cells in humans, which is mainly through apoptosis (Paris et al., 2004), lipid metabolism (Rakotomanga et al., 2007), and immunomodulatory effects (Liu et al., 2009; Wadhone et al., 2009). However, this drug also has the drawback of renal toxicity and is teratogenic, and not suitable to treat pregnant women. **1e** analogs included two series of ether phospholipids: 1) cyclohexylidene or cyclopentadecylidene substituted ether phospholipids with *N,N,N*-trimethylammonium or *N*-methylpiperidino or *N*-methylmorpholine head groups and 2) rigid head groups in combination with cycloalkylidene moieties in the lipid portion were reported to be 1.5 to 62 times more potent than miltefosine (Calogeropoulou et al., 2008). An alkyl phosphocholine-dinitroaniline hybrid molecule was reported to be effective against promastigotes and intracellular amastigotes form of *L. amazonensis* and more selective than miltefosine (Godinho et al., 2013)*.* Another study showed that a new series of 2-[3-(2-alkyloxy-ethyl)-adamantan-1-yl]-ethoxy substituted ether phospholipids possessed antiparasitic activity with respect to the 2-alkyloxy substituent and were less cytotoxic compared to miltefosine (Papanastasiou et al., 2010).

### Emerging Drug Targets and Drugs Against Leishmaniasis

#### Nucleoside Analogs and Purine Salvage Pathway as Drug Targets

The parasitic protozoan *Leishmania* lacks enzymes that are necessary for *de novo* synthesis of purine nucleotides. Usually, they are compensated by obtaining purine bases from the mammalian host cells *via* purine salvage system. Here, nucleoside transporters play vital roles in translocating purine bases through the cell surface of parasites.

The parasitic transporters are found to be unique compared to the transporters in the mammalian cells, in terms of substrate specificity. There are various pathways for nucleoside uptake with several purine/pyrimidine transporters involved such as LdNT1 and LdNT2, which are well studied (Vasudevan et al., 1998; Carter et al., 2000). Selectively targeting these transporters is difficult but it still remains a potential target, as they can uptake even toxic analogs of nucleosides that inhibit the cell growth of parasites.

**FIGURE 2:**
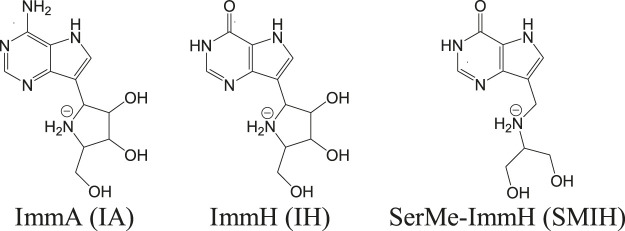
Immucillines, the nucleoside analogs as hit candidates against leishmaniasis.

Apart from these transporters, there are significant enzymes in the purine salvage pathway that include phosphoribosyl transferases. Purine analogs like allopurinol are known to inhibit phosphoribosyl transferases and are effective against parasites (Martinez and Marr, 1992). Allopurinol is effective against canine leishmaniasis but poses pharmacokinetic safety issues in case of humans (Yasur-Landau et al., 2016). *In vitro* studies have revealed a reduction in intramacrophagic amastigotes of *L. infantum* with the use of nucleoside analogs like immucillins (Freitas et al., 2015a). Immucillins or the synthetic deazapurine nucleoside analogs that include ImmA (IA), ImmH (IH), and SerMe-ImmH (SMIH) and their respective chemical structures are given in Figure 2 (Freitas et al., 2015b).

Another important enzyme in the purine salvage pathway includes the nucleoside diphosphate kinases (NDKs). An analog of a multitargeted receptor tyrosine kinase (RTK) inhibitor (Chow and Eckhardt, 2007) and a pyrrole-indolinone compound (Vieira et al., 2017) was reported, which binds NDK of *L. major* and exhibits antileishmanial activity *in vitro.* The latter drug was reported to possess potency and efficacy similar to that of the drug amphotericin B. The authors have suggested the use of this compound as a scaffold to develop new inhibitors against NDKs of *Leishmania* spp. (Vieira et al., 2017).

Though pyrimidine is synthesized by both *de novo* and through pyrimidine salvage pathway, enzymes of this pathway as well as the pyrimidine analogs are regarded as promising drug targets against *Leishmania* (Alzahrani et al., 2017). Recently, *in vitro* studies revealed the use of pyrimidine analogs, cytarabine, and 5-fluorouracil and two purine analogs, azathioprine and 6-mercaptopurine, against the growth of promastigote and amastigote forms of *L. donovani and L. infantum* (Azzouz and Lawton, 2017). Out of these four analogs, cytarabine and 5-fluorouracil were efficient against the promastigote stage of parasites and 5-fluorouracil and azathioprine were effective against the intracellular amastigotes. Here 5-fluorouracil was found to be highly efficient against both stages of parasites by inducing cytoplasmic vacuolization, causing damage to mitochondria, and subsequently damaging the kinetoplast and death of the parasites.

#### Kinetoplastid Proteasome Inhibition

**FIGURE 3:**
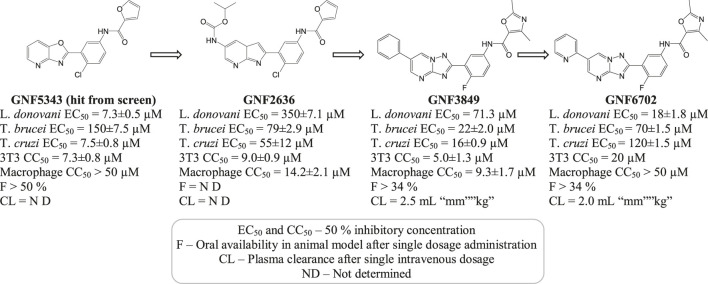
Chemical evolution of proteasome targeting triazolopyrimidine scaffold inhibitors that act by inhibiting the activity of chymotrypsin protease.

A selective (20S) proteasome inhibitor GNF6702 (Khare et al., 2016) that has evolved through phenotypic hit has proved proteasomes as a promising target for the treatment of infections by kinetoplastid parasites (Crunkhorn, 2016) including *T. cruzi, Leishmania* species, and *T. brucei*. The triazolopyrimidine scaffold of GNF6702 exhibited antiparasitic activity by inhibiting chymotrypsin protease activity. Figure 3 depicts the evolution of triazolopyrimidine scaffold drugs. The mode of action of this drug was known to occur by noncompetitive mode, without hindering the mammalian proteasomes or cells.

**FIGURE 4:**
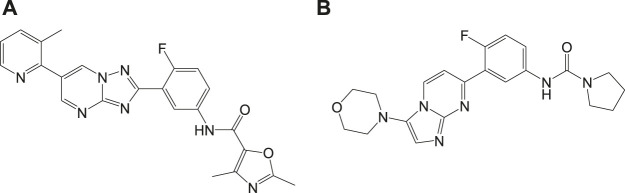
Compounds that inhibit β5 subunits of proteasome.

Recently, a structurally related proteasome inhibitor, **4a** (LXE408) which is at present in phase 1 human clinical trials (Nagle et al., 2020) was reported. A novel mode of noncompetitive binding drug compound **4a** with specific β5 proteasome was also reported (Figure 4). Oral administration of compound **4a** was reported to have high efficacy in infected mouse models in its preclinical studies and is currently tested for its safety and tolerability in phase I clinical trials.

Another potential antileishmanial compound **4b** (Wyllie et al., 2019) was reported in the literature and its chemical structure is shown in Figure 4. The activity of this compound was verified against different clinically significant parasites like *L. donovani*, *L. infantum*, etc. This compound was reported to possess good pharmacokinetics, *in vivo* efficacy in infected mouse models, and comparable efficacy to the only approved oral antileishmanial drug miltefosine. The mechanism of action of this drug involves the profound inhibition of β5 subunit of the proteasome *via* chymotrypsin-like activity in parasites. With consistent experimental data and high-resolution cryo-EM studies on this compound-proteasome complex, a new inhibitor site located between β4 and β5 proteasome subunits was reported. Due to the positive results in regard to safety and efficacy, this compound is taken further for human trials.

#### Other Targets and Inhibitors Under Investigation

Mitochondria, being one of the most important organelles for parasites, with their unique properties and proteins that differ from mammalian hosts, serve as a potential target for the development of therapeutics against parasitic infections like leishmaniasis (Tasbihi et al., 2019). Currently, the studies on mitochondria of parasites are limited; however, with the available information, chemotherapeutic approaches are being developed against the mitochondria of parasites (Villa-Pulgarín et al., 2017). For instance, chalcones are reported as potential lead compounds against leishmaniasis which target the mitochondrial structure (Zhai et al., 1995; Zhai et al., 1999) and function in the parasites, followed by the inhibition of fumarate reductase (Chen et al., 2001).

Mitochondrial cytochrome bc 1 plays a vital role in electron transport chain and is reported as a potential drug target. Endochin-like quinolones (ELQs) were studied to show toxicity against amastigote forms of *L. donovani* and *L. mexicana* targeting cytochrome bc 1 (Stickles et al., 2015; Ortiz et al., 2016). Hydroxynaphthoquinone buparvaquone has also been reported to be active against cytochrome bc 1, which inhibits electron transport, ATP synthesis, and parasite survival.

**FIGURE 5:**
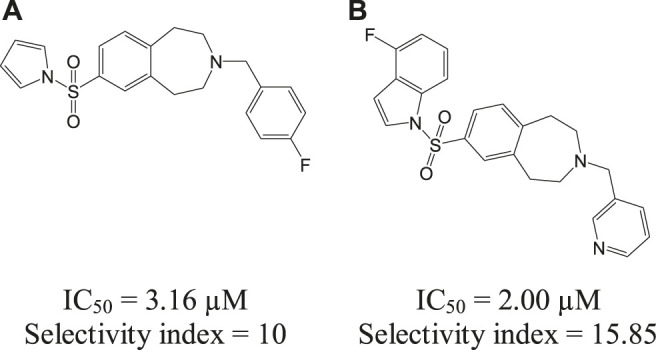
Benzazepane-based hit compounds that act as inhibitors by targeting *Leishmania* inositol phosphorylceramide synthases.

The *Leishmania* inositol phosphoryl ceramide (IPC) synthases (Norcliffe et al., 2018) are regarded as one of the potential drug targets. Modeling studies have revealed coumarin derivatives to possess antileishmanial activity, which were further validated by *in vivo* studies (Mandlik et al., 2016; Mandlik and Singh, 2016). *In vitro* studies with a new class of benzazepanes (**5a** and **5b**) are shown in Figure 5. Compounds **5a** and **5b** were reported to exhibit anti-*Leishmania* IPC synthase activity (Norcliffe et al., 2018). Similar compounds could be developed and studied to obtain new antileishmanial drugs. Apart from these, kinetoplastid topoisomerases (Das et al., 2008) and nuclear DNA primases (Bhowmik et al., 2020) of *Leishmania* species are suggested as other potential drug targets, for their structural diversity from mammalian hosts as well as their significance in DNA replication in the parasites.

**FIGURE 6:**
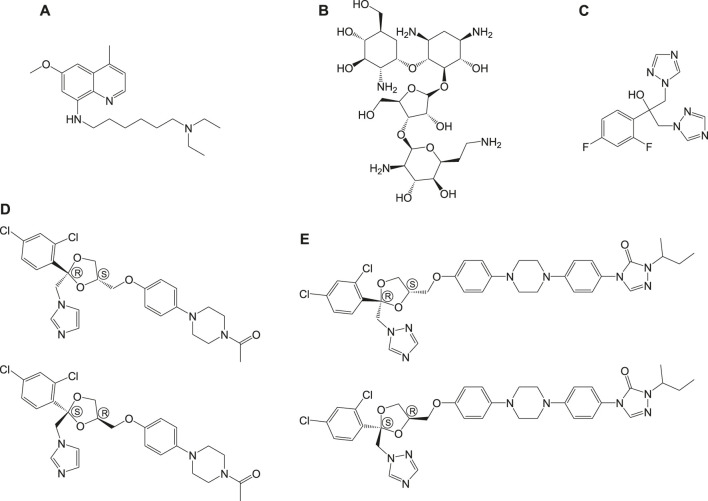
Chemical structure of anti-leishmaniasis hit compounds.

Sitamaquine (**6a**, Figure 6) (8-aminoquinolines) (Yeates, 2002) is an antileishmanial oral drug, in which the molecular mechanism of action is yet to be understood. Although sitamaquine is reported to cause visual morphological changes in the parasite (LANGRETH et al., 1983), the specific target is yet to be identified. The interaction of sitamaquine with *L. donovani* revealed that the rapid diffusion of drug through the membrane accumulates in the cytosol by a process independent of energy or sterol in the parasite (Coimbra et al., 2010). Also, the interaction of **6a** with the parasite membrane is transitory (Dueñas-Romero et al., 2007), and its efflux requires energy.

*Paromomycin* (**6b**, Figure 6) in the form of topical formulations was reported for leishmaniasis treatment. Paromomycin (paromomycin sulfate, C_23_H_47_O_18_S) drugs are aminoglycoside antibiotics, administered intramuscularly, to treat leishmaniasis in which mode of action is reported to involve mitochondria (Maarouf et al., 1997) and ribosomes (Maarouf et al., 1995) causing protein inhibition in the parasite. Another group of compounds used for the treatment of leishmaniasis are referred to as the *azoles*, for example, fluconazole (**6c**), ketoconazole (**6d**), and itraconazole (**6e**) as given in Figure 6 (Prates et al., 2017; Vera et al., 2018; Biswaro et al., 2019). However, the use of azoles was reported to show mixed results (Nagle et al., 2014; Galvao et al., 2017).

The enzymes, bifunctional dihydrofolate reductase-thymidylate synthase, and pteridine reductase 1 in *Leishmania* spp. that are involved in the folate metabolic pathway were studied as targets for leishmaniasis treatment (Docampo et al., 2011). Dihydrofolate reductase enzyme was reported to be unique in the protozoan parasite *Leishmania* relative to that in the human host, thereby considering the enzyme as a promising target for selective drug design (Gilbert, 2002). In addition, pteridine reductase 1 inhibition was found to be essential to avoid the resistance to dihydrofolate reductase inhibitors. Chroman-4-one scaffold was reported to be a promising scaffold for the development of pteridine reductase 1 inhibitors against *Leishmania* spp., with low toxicity (Di Pisa et al., 2017). Structure-based optimization of piperidine-pteridine derivatives resulted in two compounds, methyl-1-(4-(((2,4-diaminopteridin-6-yl)methyl)(2-ethoxy-2-oxoethyl)amino)benzoyl)piperidine-4-carboxylate and methyl 1-(4-(((2,4-Diaminopteridin-6-yl)methyl)(2-hydroxyethyl)-amino)benzoyl)piperidine-4-carboxylate, with relatively high potency, enhanced binding affinity, and selectivity among other derivatives (Corona et al., 2012). Novel derivative compounds of arylnicotinic acids conjugated with aryl (thio)semicarbazides were also reported to be synthesized based on structure-guided approach targeting the enzyme pteridine reductase 1 (*L. major*) which was also proven to possess enhanced selectivity and antiamastigote activity relative to miltefosine (Eldehna et al., 2019).

#### Liposomal Encapsulation and Nanoparticles in Anti‐leishmanial Drugs

Nanotechnology-based drug delivery or drug formulations were attempted to improve the efficacy and also to assure safety of drugs. The challenges in oral route of administration of pentamidine drugs were reported to be overcome by means of loading the drug in PLGA nanoparticles (Valle et al., 2019). Here, the nanoparticles were reported to be prepared by double emulsion method and *in vitro*/*in vivo* studies (infected BALB/c mice) were successful. These drug-loaded PLGA nanoparticles have shown a new perspective to drug administration with good pharmacological activity. Also, high efficacy has been reported with nanoformulations of amphotericin B (Manandhar et al., 2014), which is more safe and cost-effective and serves as an alternative to the existing conventional drug **1d**. Topical nanoliposomal formulations were prepared to treat cutaneous leishmaniasis, with liposomes containing 0.1, 0.2, and 0.4% **1d** (Lip-AmB), out of which 0.4% Lip-AmB was found to possess longer stability for about 20 months at room temperature (Jaafari et al., 2019). These formulations were effective against the lesions in *L. major*-infected *BALB/c mice* and also showed inhibition of the promastigotes/amastigotes growth *in vitro.* Fungisome is a liposomal formulation marketed in India (Wijnant et al., 2018), with lipids and formulations different from that of the standard AmBisome. In a study with *L. major*-infected BALB/c murine model (cutaneous leishmaniasis infection), it was reported that fungisome at a dosage of 1 mg/kg revealed toxicity, whereas AmBisome was nontoxic. However, the antileishmanial activity was exhibited with ∼5–10 mg/kg of fungisome and also proved to be less efficacious when compared with AmBisome.

*Artemisinin* and its derivatives are another group of molecules that are reported to be effective for visceral leishmaniasis treatment. These are found to work by generating free radicals leading to apoptosis in parasites (Sen et al., 2010). *In vivo* studies in mice with nanoliposomal formulations of artemisinin were reported to reduce leishmanial intracellular infections (Ghaffarifar et al., 2015). Polymer (PLGA-poly (d,l-lactic-co-glycolic acid))-based nanoparticles were also shown to act as carrier of the drug artemisinin, facilitating sustained release of the drug (*in vitro* studies) along with antileishmanial activity (*ex vivo*). These artemisinin-incorporated PLGA polymeric nanoparticles also exhibited reduction of parasites or antiparasitic activity (Want et al., 2014; Want et al., 2015; Want et al., 2017). The pros of using these drug-loaded nanoparticles include reduction of drug toxicity, enhanced hydrophilicity, and bioavailability. However, these nanoparticles have not attained the level of clinical trials due to the higher cost, lower drug load, and toxicity involved in the preparation stage. Also, the limited availability of reports on nanoparticles with antileishmanial activity necessitated the need for exploration in this field of research that can reach clinical trials.

A recent *in vitro* study involved the use of an FDA-approved drug for malaria treatment (Chavali et al., 2012), halofantrine, for leishmaniasis treatment. By liposomal encapsulation of halofantrine, called halolipo, the issues of hydrophobicity and cardiotoxicity of the halofantrine drug were overcome. Halolipo (∼20 nm diameter), on exposure to the protozoan parasite *L. donovani* (promastigotes), led to reduction in viable number of parasites. This could be due to ROS generation and depolarization of mitochondrial membrane of the parasites. Also, *in vitro* studies on the same confirm the selective reduction of viable parasites, dependency on drug concentration, and nontoxicity to mammalian host cells. However, *in vivo* studies ought to be performed with halolipo, so that this drug could be developed as an effective therapeutic for leishmaniasis in near future.

Use of nanosilver has also been tested by several researchers as therapeutics for leishmaniasis. It was reported to possess higher potency as the size becomes smaller (Allahverdiyev et al., 2011a; Allahverdiyev et al., 2011b). Nanosilver is shown to cause DNA damage and generate free radicals in the parasites with increase in immunomodulatory effects in human cell lines (de Menezes et al., 2015). Selenium nanoparticles known to possess antimicrobial, anticancerous, and antioxidant properties were used to develop a novel approach to treat cutaneous lesions and were proven to exhibit antileishmanial activity in both *in vivo* and *in vitro* studies. Also, these nanoparticles exhibited significant potency against *L. tropica* (Shakibaie et al., 2015). Another interesting finding involved silver-doped titanium oxide (TiAg), formulated by green synthesis using essential oil from *Nigella sativa* which showed high antileishmanial activity against *L. tropica* and *L. infantum* (Allahverdiyev et al., 2013). This formulation was proven to be nontoxic with high potency for treatment of cutaneous leishmaniasis. Another nanoformulation based on the combination of the miltefosine drug (Figure 1) with curcumin nanoformulation was reported (Tiwari et al., 2017) to possess enhanced antileishmanial activity both *in vitro* and *in vivo*.

## Chagas Disease

Chagas disease, also called American trypanosomiasis, is one of the NTDs, which is endemic mainly in Latin America and spreads in European countries, Australia, Japan, Canada, and southern parts of the United States. Chagas disease is caused by a parasite named *T. cruzi*, a hemoflagellate protozoan of Kinetoplastida order and Trypanosomatidae family, which can occur in two phases, acute and chronic. This disease was discovered by a physician Carlos Chagas, who had stated during the initial period that there was no specific treatment for this disease, in his publication “Manual of Tropical and Infectious Diseases.” In Chagas disease, the causative protozoan is *Trypanosoma cruzi*, from which the trypomastigote forms are transmitted to humans through insects that belong to the subfamily Triatominae. These infective trypomastigotes are present in the fecal matter of the bug and enter into the tissues of human host through the bitten wound and later develop into an intracellular replicative parasitic form of amastigotes. Other modes of transmission are through infected blood transfusion/organ transplantation and genetic transmission from mother to fetus. Chagas disease occurs mainly through two routes, congenital and oral. The congenital Chagas disease occurs throughout the world even in the nonendemic areas. The oral Chagas disease leads to acute disease outbreaks in the areas facing vectorial route interruptions (Sánchez and Ramãrez, 2013). Congenital Chagas disease is mostly asymptomatic but includes some nonspecific clinical manifestations like myocarditis, gastrointestinal problems, meningoencephalitis, anemia, low birth weight, jaundice, microencephaly, ocular lesions, etc. (Schijman, 2006; Punukollu et al., 2007). Around 30% of these congenitally affected patients tend to develop symptomatic chronic phase of Chagas disease with cardiac and digestive problems (Rassi et al., 2010; Carlier et al., 2011). These patients possess lower possibility of developing cardiac problems relative to that in patients infected by vectorial route (Bern et al., 2011; Fabbro et al., 2014). In patients with oral Chagas disease infection, high mortality rate is observed in the first two weeks after infection followed by inflammation in the gastric mucosa which extends to proximate mesentery (Hoft et al., 1996). Clinical manifestations like myocarditis, acute heart failure, and meningoencephalitis are observed with orally infected Chagas disease (Punukollu et al., 2007; Shikanai-Yasuda and Carvalho, 2012). In case of acute infections through oral route, clinical features include abdominal pain, gastrointestinal tract bleeding, heart murmurs, palpitations, jaundice, nausea, vomiting, hepatomegaly, enteritis, and chest pain (WHO-Expert-Committee, 2002; Andrade et al., 2014). During the 1960s, the therapeutic activity of nitrofurans in infected murines and humans was discovered but it had a low curative effect which stopped its further development. Moreover, their curative ability differed in geographic areas, potentially due to mutations/variations in genetic strains of the parasites. These drugs were not effective against chronic cases of Chagas disease and include several serious side effects like anorexia, digestive manifestations, hypersensitivity, bone marrow depression, dermatitis with cutaneous eruptions, and peripheral polyneuropathy. The beginning of the 1980s marked the introduction of techniques like immunohistochemistry and PCR, which confirmed the association of parasites and their DNA with the inflammatory reactions resulting in pathological lesions in chronic Chagas disease. Several experimental studies (Morillo et al., 2015; Soy et al., 2015; Fernández et al., 2016) and clinical trials (Andrade et al., 2013; Chatelain, 2017) have been reported to show the significance of reducing the parasite load in chronic Chagas disease treatment and the associated chronic cardiomyopathy.

### Standard Anti‐chagasic Drugs

**FIGURE 7:**
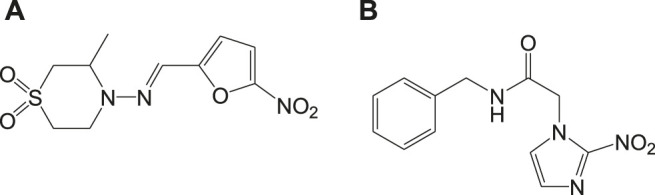
Chemical structure of antichagasic nitro heterocyclic compounds.

At present, Chagas disease is treated by the two nitro heterocyclic compounds that were discovered in 1960–1970, namely, nifurtimox (**7a**) and benznidazole (**7b)** (Maya Arango et al., 2010), and the chemical structures are shown in Figure 7.

These drugs work by accumulation of free radicals generated by means of the nitro groups, which further resulted in high antioxidant activities in *T. cruzi,* ultimately killing the parasites. As stated before, these drugs are effective only for acute cases of Chagas disease infection along with side effects.

### Emerging Drug Targets and Drugs Against Chagas Disease

#### CYP51 Inhibitors

**FIGURE 8:**
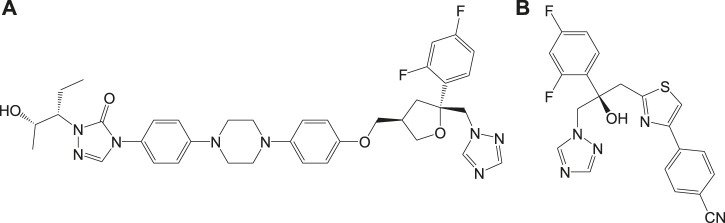
Potent anti-Chagas azole-derived compounds that target sterol biosynthetic pathway.

Inhibitors of sterol biosynthetic pathway have been reported as one of the new drug targets for the treatment of Chagas disease. In particular, C14-ademethylase (CYP51) represents a potential drug target in which various inhibitors like azoles block the ergosterol biosynthesis and sterol C14-demethylase (CYP51) activities in the parasite. *In vivo* studies have revealed that the above compounds possess high potency of antichagasic activity along with good pharmacokinetic properties like longer half-life and large volume of distribution as compared to the previously existing drugs. Phase II clinical trials of the drugs (Figure 8), posaconazole (**8a**), and a prodrug of ravuconazole (**8b**) were reported to ensure safety, without sustained efficacy as single medications, in patients with chronic Chagas disease (Tarleton et al., 2014; Álvarez et al., 2015).

The other strategies involved variation in dosages and combination of drugs to improve the efficacy against Chagas disease. It was also reported that prolonged treatment with azole drugs like **8a** is required for potent antichagasic activity. This idea is also validated by Guedes et al. (da Matta Guedes et al., 2004) in infected dogs with treatment using benznidazole or albaconazole which resulted in complete cure within 60 days of treatment and 90 days of treatment, respectively. This necessitates the consideration of individual dosage regimens, drug concentration, and treatment time for successful treatment. With the high efficacy of azole derivatives, researchers are currently working on developing rational approaches for antichagasic therapeutics.

**FIGURE 9:**
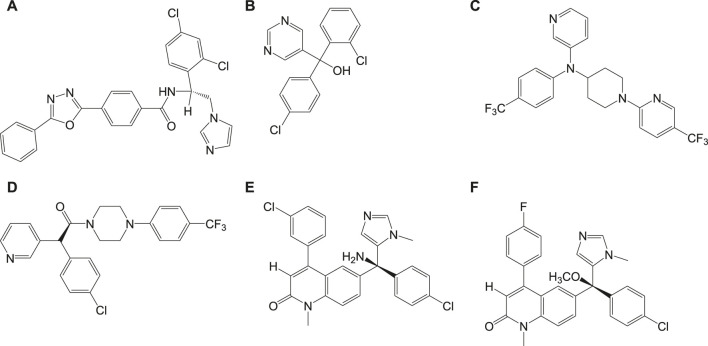
Potent anti-Chagas nonazole-derived drugs that target sterol biosynthetic pathway.

*In vivo* studies with nonazole derivative **9a** for CYP51 inhibition have revealed variations in antichagasic activity with different parasitic strains. With a nonazole CYP51 inhibiting compound, fenarimol (**9b**) as scaffold, two other lead compounds **9c** and **9d** were developed which were reported to possess antichagasic activity similar to that of **8a** and better than **7b** (Keenan et al., 2013). Buckner et al. developed an analog of tipifarnib (**9e**), anticancer drug, in which farnesyltransferase inhibitory activity was eliminated and CYP51 inhibition was withheld (Buckner et al., 2012). This compound was found to be highly potent with antichagasic activity in mouse models but requires improvement in pharmacokinetic properties. Similarly, another compound **9f**, an atropisomer evolved from **9e**, was found to possess similar potency against *T. cruzi*. The chemical structures of nonazole-derived drugs that target the sterol biosynthetic pathway are shown in Figure 9.

#### Cruzain Inhibitors

**FIGURE 10:**
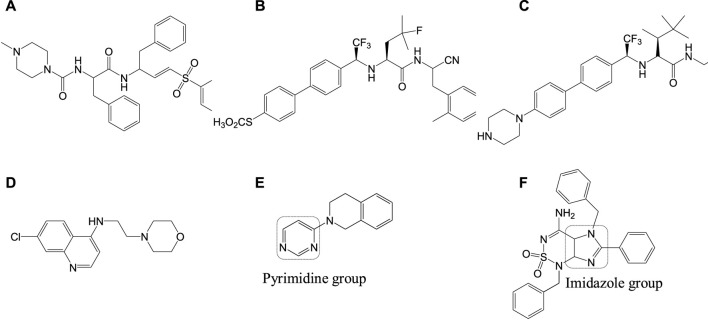
Anti-Chagas drugs that inhibit cathepsin L-cysteine protease.

Cruzipain or cruzain denotes cathepsin L-cysteine protease that performs proteolytic activity throughout all stages in the life of *T. cruzi*. Studies have reported the importance of this enzyme in various biological functions including evasion of the immune system and host-parasite interactions (Brak et al., 2010; Bahia et al., 2014). Inhibitors of this protease also serve as significant drug targets in Chagas disease therapeutics. Compound **10a**, a vinyl sulfone, was rationally designed to selectively inhibit this cruzipain protease. This compound was reported to exhibit anti-*T. cruzi* activity in experimental mice models (immunocompetent and immunodeficient) infected with *T. cruzi* (Doyle et al., 2007). However, complete curability was not observed with compound **10a** when tested on acutely infected (*T. cruzi*) dog models, but it was reported to reduce the myocardial damage (Barr et al., 2005). Compound **10a** is currently in its advanced stages of preclinical studies, and also several other cruzipain inhibitor compounds with better potency and selective anti-*T. cruzi* activities are being developed (Brak et al., 2010; Ndao et al., 2014). Recently, two compounds **10b** and **10c** were identified by Ndao et al., which showed curability of 78 and 90%, respectively, in acutely infected murines (Ndao et al., 2014). Several analogs of 8-chloro-N-(3-morpholinopropyl)-5H-pyrimido [5,4-b]indol-4-amine containing indole, pyrimidine, quinoline, aniline, and pyrrole groups were reported to be tested for antichagasic activity. Among the several derivatives, 4-aminoquinoline analogs, in particular compound **10d**, were reported to have anticruzain activity along with antichagasic activity against *T. cruzi* (Tulahuen strain) with lower selectivity. Pyrimidine analog **10e** was found to be highly active and selective for Chagas disease but lacked anticruzain activity (Schmidt and Krauth-Siegel, 2002). This implicates the need to understand the mechanism of action behind these compounds with *in vivo* studies (Arias et al., 2017; Vázquez et al., 2017). Also, several computational screening studies have revealed the synthesis and biological activities of several imidazole compounds. Among the various compounds identified, compound **10f** (Rodrígues-Poveda et al., 2012) was shown to exhibit high anti-*T. cruzi* activity against three strains including Tulahuen 2, CL-clone B5, and Y. The structures of potent inhibitors that target cathepsin L-cysteine protease are given in Figure 10.

#### Mitochondrial Deregulation Drugs

**FIGURE 11:**
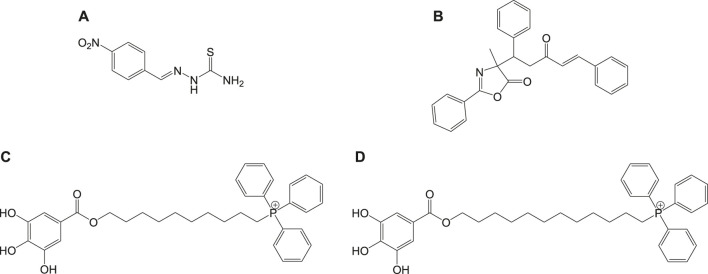
Potent anti-Chagas drugs that variously deregulate mitochondrial functions.

Mitochondria are vital cell organelles that are involved in ATP synthesis, nutrient oxidation, cellular redox, calcium homeostasis, and apoptosis. It produces hydrogen peroxide as the main oxidative species for cellular signaling in cytosol, and deregulation of mitochondria causes an increase in reactive oxygen species, leading to cytotoxicity and cell death. *T. cruzi* possesses unique mitochondria containing kinetoplasts and compactly packed mitochondrial DNA that accounts for 30% of its entire genome, making mitochondria an important drug target for Chagas disease treatment. An *in vitro* study has investigated the antichagasic activity of 4-nitrobenzaldehyde thiosemicarbazone (**11a**) (Figure 11), an S-limonene derivative, which showed potent activity against trypomastigotes and amastigotes of *T. cruzi*. Compound **11b** was reported to deregulate mitochondria of the parasites by cytoplasmic vacuolization, reduction in membrane potential, and increased free radical generation (Britta et al., 2015). Another study evaluated four C-4 functionalized azalactone derivatives for antichagasic activity (de Azeredo et al., 2017). Among these derivatives, one compound **11c** was found to possess three times more anti-*T. cruzi* activity compared to that of the antichagasic drug **7b**. Reduction in mitochondrial membrane potential and size of epimastigotes was observed by flow cytometry and electron microscopy studies. It is recommended that these compounds could be used as a scaffold in the development of new therapeutic drugs for Chagas disease (de Azeredo et al., 2017). Yet another antichagasic agent was developed using gallic acid derivatives possessing lipophilic groups and triphenylphosphonium moiety that target the mitochondria of *T.* cruzi. Among these derivatives, relatively more potent compounds **11c** and **11d** compared to compound **7a** were reported (Cortes et al., 2015). These compounds were also highly selective and formed pores on mitochondrial membrane by varying the membrane potential. Further new analogs with antichagasic activity can potentially be developed from these derivatives.

#### Trypanothione Reductase

Trypanothione reductase is another significant therapeutic target for Chagas disease treatment, mainly because of its unique presence in Trypanosomatidae family and not in mammalian cells (Krieger et al., 2000; Khan, 2007; Beig et al., 2015). The reduction of trypanothione disulphide to trypanothione occurs in the presence of NADPH and is catalyzed by the enzyme trypanothione reductase (Fairlamb et al., 1985; Bond et al., 1999; Krieger et al., 2000; Beig et al., 2015).

**FIGURE 12:**
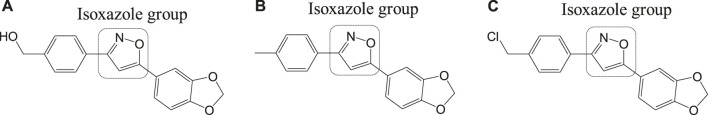
Potent anti-Chagas isoxazole-derived drugs that target trypanothione reductase.

Different isoxazole analogs were reported to be synthesized by microwave irradiation, with structures based on the natural lignans, veraguensin, and grandisin which exhibited potent activities against *T. cruzi* (Tulahuen strain) trypomastigote forms in the circulating blood and amastigote forms within the cells (da Rosa et al., 2017). Three compounds **12a**, **12b**, and **12c** (Figure 12) were found to be potent against amastigotes at concentrations lower than that reported for benznidazole derivatives but were inactive against trypomastigotes. By means of enzymatic assays, isoxazoles were reported to act independently on the enzyme trypanothione reductase. Recent studies have reported computationally screened and evaluated trypanothione reductase inhibitors against Chagas disease, which were obtained from natural products database (da Paixão and da Rocha Pita, 2019).

#### Heme Peroxidation as Drug Target

**FIGURE 13:**
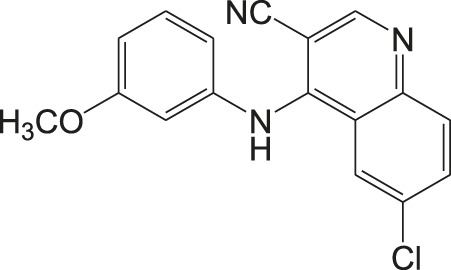
Quinoline derivative that inhibits heme peroxidation by forming heme-quinoline complex as potent antichagasic lead candidate.

In general, heme or ferroprotoporphyrin plays a significant role in various biological processes including energy production, detoxification, respiration, oxygen transport, and antioxidant gene expression. Breakdown of heme is known to generate free radicals, which causes damage to DNA, proteins, lipids, and ultimately the cells. The requirement of heme in *T. cruzi* for multiplication of epimastigote forms by redox mechanism is stated as a potential drug target that can be blocked to possibly inhibit the epimastigotes (de Almeida Nogueira et al., 2011; Nogueira et al., 2017). Quinoline compounds are made of nitrogen heterocyclic groups that form complexes with heme and have been reported to possess activity against plasmodium, *Leishmania*, *T. cruzi*, bacteria, and also cancer (Eswaran et al., 2010; Ganguly et al., 2011; Muscia et al., 2011). Several derivatives of 4-arylaminoquinoline-3-carbonitrile were reported to exhibit anti-*T. cruzi* activity; in particular, compound **13** was found to be more active than compound **7b**, when complexed with hemin (Lechuga et al., 2016). The authors had reported enzymatic conversion of heme to biliverdin favoring oxygenase activity in parasites and also the complex formation of heme-quinoline compound **13** (Figure 13) leading to a reduction in heme levels followed by the generation of reactive oxygen species resulting in the death of parasites.

#### Other Targets and Formulations for Anti-Chagas Drug Development

*T. cruzi* depends on inherent sterols including ergosterol and its analogs for survival, where ergosterols play an important part in maintaining the plasma membrane (Urbina et al., 2002; Shang et al., 2014). Ergosterol requires squalene synthase for its biosynthesis, which catalyzes squalene formation by dimerization of two farnesyl pyrophosphate (FPP) molecules (Urbina et al., 2002). Researchers have found that inhibition of the squalene synthase can be a potential drug target for Chagas disease treatment. Inhibitors targeting squalene synthase were developed by several researchers (Chao et al., 2017) and are still in the process of investigation. Similarly, farnesyl diphosphate synthase (FPPS) in the mevalonate pathway is stated as a potential target for several parasitic diseases (Demoro et al., 2012; Ferrer-Casal et al., 2014). Inhibition of FPPS indirectly blocks the synthesis of farnesyl diphosphate, by blocking the synthesis of farnesyl pyrophosphate and geranylgeranyl pyrophosphate (Aripirala et al., 2012). Several bisphosphonate derivatives are under research against *T. cruzi* by FPPS inhibition, to obtain new therapeutic drugs for Chagas disease (Recher et al., 2013). *T. cruzi* contains four iron superoxide dismutases (Fe-SODs) formed in the mitochondria (TcSODA and C), cytosol (TcSODB1), and glycosomes (TcSODB1-2) (Piacenza et al., 2013), which are unique to these parasites and different from that in humans, thereby serving as potential targets (Mateo et al., 2008). These superoxide dismutases are known to neutralize toxicity generated by oxygen radicals (Temperton et al., 1998). Studies evaluating the antichagasic activity of potential drugs by inhibition of Fe-SOD were also reported; for instance, a series of derivatives of arylaminoketones were tested (Moreno-Viguri et al., 2016) and shown to exert antichagasic activity by means of Fe-SOD inhibition. It is confirmed by analyzing the 1H-Magnetic Nuclear Resonance (^1^H NMR) chemical shifts of excreted metabolites from the cultures of epimastigotes of *T. cruzi Arequipa* strain that was treated with test compounds.

Nanostructure formulations with trypanocidal activity were developed to lower the toxicity of drugs like **7b**. Nanoparticles including nanostructured lipid carriers, liposomes, quatsomes, solid lipid nanoparticles, and cyclodextrins were evaluated (Scalise et al., 2016; Vinuesa et al., 2017). Cyclodextrins-benznidazole complexes were reported to be relatively lower than that of benznidazole in its free form (Vinuesa et al., 2017).

## Conclusion

Although the spread of both leishmaniasis and Chagas disease is under control, the threat still exists because of limited therapeutics and increasing incidents of drug resistance. Many drugs that are available to treat these diseases possess serious side effects and existing diversity among the causing parasites. Moreover, many drugs are active only in the acute phase of the disease. Herein, the various therapeutic strategies, drug targets, and drugs for leishmaniasis and Chagas disease were discussed. The significance of these two diseases based on the economic burden and the disability caused by them has opened new avenues for novel therapeutics and drug targets. The presently employed strategies like novel drug developments and the use of a combination or repurposed drugs have been discussed briefly besides their advantages and future scopes. For leishmaniasis, standard drugs like pentavalent antimonials, pentamidine, amphotericin B, and miltefosine that target various metabolic pathways are discussed with their mechanism of action, advantages over other drugs, and limitations on their use. Various drugs that target the purine salvage pathway, purine and pyrimidine analogs, and their efficiencies at various stages of the pathogens cycle are also reviewed. The role of nanotechnology, various proteasome inhibitors, and enzymes that are under investigation as potential targets for leishmaniasis are also reviewed. With Chagas disease, various standard drugs like nifurtimox and benznidazole and their mechanism of action towards killing the parasites are discussed. Various inhibitors for Chagas disease that target CYP51, cruzain, trypanothione reductase, mitochondrial functions, heme peroxidation, etc. are also discussed. The recently developed nanostructure formulations including nanostructured lipid carriers, liposomes, quatsomes, solid lipid nanoparticles, and cyclodextrins that can reduce the toxic effects of anti-Chagas drugs are detailed.

This review details the advancements towards drug development. In recent years, it has been observed that the sensitivity of the available standard drugs varied in clinical samples over years, due to acquired resistance. Therefore, new therapies and strategies also need to be identified and implemented to prevent the emergence of new drug-resistant strains. Combination therapies and improved diagnosis could play key roles in disease management approaches.
